# Lipases Immobilization for Effective Synthesis of Biodiesel Starting from Coffee Waste Oils

**DOI:** 10.3390/biom3030514

**Published:** 2013-08-13

**Authors:** Valerio Ferrario, Harumi Veny, Elisabetta De Angelis, Luciano Navarini, Cynthia Ebert, Lucia Gardossi

**Affiliations:** 1 Dipartimento di Scienze Chimiche e Farmaceutiche, Università degli Studi di Trieste, Piazzale Europa 1, Trieste 34127, Italy; E-Mails: vferrario@units.it (V.F.); ebert@units.it (C.E.); 2 Department of Chemical Engineering, Faculty of Engineering, University of Malaya, Malaysia; E-Mail: my_harumi@yahoo.com (H.V.); 3 illycaffè S.p.A., via Flavia 110, Trieste 34147, Italy; E-Mails: elisabetta.deangelis@illy.com (E.D.A.); luciano.navarini@illy.com (L.N.)

**Keywords:** lipases, immobilization, biodiesel, oil from spent coffee ground

## Abstract

Immobilized lipases were applied to the enzymatic conversion of oils from spent coffee ground into biodiesel. Two lipases were selected for the study because of their conformational behavior analysed by Molecular Dynamics (MD) simulations taking into account that immobilization conditions affect conformational behavior of the lipases and ultimately, their efficiency upon immobilization. The enzymatic synthesis of biodiesel was initially carried out on a model substrate (triolein) in order to select the most promising immobilized biocatalysts. The results indicate that oils can be converted quantitatively within hours. The role of the nature of the immobilization support emerged as a key factor affecting reaction rate, most probably because of partition and mass transfer barriers occurring with hydrophilic solid supports. Finally, oil from spent coffee ground was transformed into biodiesel with yields ranging from 55% to 72%. The synthesis is of particular interest in the perspective of developing sustainable processes for the production of bio-fuels from food wastes and renewable materials. The enzymatic synthesis of biodiesel is carried out under mild conditions, with stoichiometric amounts of substrates (oil and methanol) and the removal of free fatty acids is not required.

## 1. Introduction

Next generation biofuels is about utilization of non-food based feedstock and more sustainable process technology. The biodiesel industrial production worldwide is predominantly by chemical transesterification from food based feedstock. This is because with chemical transesterification the highest yield can be achieved in a relatively short reaction time. However, the current major cost of biodiesel production is from the feedstock [1]. Therefore, various non food feedstocks, such as waste oil and non edible oil, have been considered and studied in view of their potential use as raw materials [2,3,4,5,6].

In most cases the chemical process is performed by an alkaline catalyst, so that acid impurities and especially free fatty acids (FFA) contained in feedstock must be removed before transesterification to avoid the formation of saponification products. For instance, a basic solution is mixed with the extracted oil so that additional pre-treatments steps are introduced in the productive cycle [7]. In this regard, lipase-catalyzed transesterification of non food feedstock is becoming more attractive for the biodiesel industry, not only because it is sustainable and environmental friendly, but also because the free fatty acids can be esterified by lipases. Recently, attention has been given to oil extracted from coffee waste and examples of chemical conversion of oil from spent coffee ground into biodiesel have been reported [8,9]. The coffee grounds are mainly composed of proteins, carbohydrates, and lipids. It must be underlined that coffee is one of the largest agricultural products and, according to the U.S. Department of Agriculture, the world’s coffee production is 16.34 billion pounds per year [8].

The spent coffee grounds contain, on an average, 15% (w/w) oil, which can be extracted by solvents such as trichloroethylene [10], *n*-hexane, ether and dichloromethane [10,11] with yields ranging from 6–28% (w/w) [9,10]. This is quite significant as compared to other major biodiesel feedstock such as rapeseed oil (37%–50%), palm oil (20%), and soybean oil (20%) [12]. Furthermore, the biodiesel from coffee possesses better stability than biodiesel from other sources due to its high antioxidant content, which hinders the rancimat process [13,14].

The work of Kondamundi reports the extraction of 15% (w/w) of oil from spent ground, which was dried prior to extraction in order to reduce the moisture content. A small amount of FFA, monoglycerides (MG), and diglycerides (DG) was also observed in the oil but a conversion in biodiesel of 100% was obtained by chemical transesterification. The oil and biodiesel formed in that process were found to be stable over one month without any observable physical changes and analysis demonstrated that biodiesel obtained from spent coffee grounds is a strong candidate as an alternative to diesel [8].

One additional issue in the development of an economically sustainable production of biodiesel by enzymatic transesterification is represented by the cost of the biocatalyst. The economic impact of biocatalysts can be reduced by immobilizing the lipases on solid supports and then recycling them. Therefore, it is important to select immobilized lipases that not only express high activity but also allow for repeated use thanks to improved stability.

Lipases are one of the classes of enzymes most largely employed in industry also because of their potential to work in non-aqueous environments [15] and they are applied at industrial scale for the transesterification of fats and oils in the food sector [16]. However, to the best of our knowledge, there is still a lack of immobilized lipases commercially available and suitable for application in biodiesel synthesis [17].

Indeed, in most cases, the lipase catalyzed synthesis of biodiesel has been studied by employing biocatalysts originally developed for interesterification of food oils [7]. However, the latter process implies the application of lipases in highly hydrophobic environments whereas biodiesel synthesis involves the use of relatively high percentages of hydrophilic short chain alcohols that have inactivating effects on enzymes [18,19]. Moreover, as the reaction proceeds, glycerol is produced. Consequently, the biocatalyst will behave quite differently in the two processes. Phenomena, such as partition of hydrophilic components, will deserve explicit attention and investigation when planning a methanolysis process.

By analyzing the immobilized lipases available on the market, Lipozyme TL IM (lipase from *Thermomyces lanuginosa*) results probably the most widely applied at industrial scale in triglyceride transformations in the food sector. It is immobilized by spraying the liquid lipase concentrate onto silica particles together with food grade granulation additives. After subsequent drying in fluid beds, the granules are ready for use in interesterification of triglycerides. However, Lipozyme TL IM will disintegrate when dispersed into water or hydrophilic media [20]. 

In the present work, the enzymatic synthesis of biodiesel was approached by studying immobilized biocatalysts specifically developed for this application. A particular attention was devoted to the nature of the immobilization carrier and the prevention of aggregation and methanol aspecific adsorption, phenomena that might cause enzyme inactivation. Finally, the selected biocatalysts were applied in the transesterification of oil extracted from spent coffee ground. Factors affecting the efficiency of lipase immobilization were also analyzed.

## 2. Results and Discussion

### 2.1. Selection of Lipases and Immobilization Supports

The present work follows our previous detailed investigation of structural and conformational properties of a series of lipases [21]. Lipases have been evolved for transforming insoluble hydrophobic substrates so that their surface present unusual features that make these proteins adapt for approaching lipophilic surfaces. Accommodation and transformation of bulky triglycerides is strictly related to the accessibility of the active site. The latter, as also the surrounding superficial area, is hydrophobic. A flexible protein domain, called lid, shields the opening of the active site when the protein is exposed to a hydrophilic environment, thus occluding the substrate access. Therefore, in principle, the immobilized lipase should maintain an open active conformation throughout the alcoholysis process despite the presence of polar hydrophilic components, such as the alcohol and the glycerol, in the reaction mixture. 

**Figure 1:**
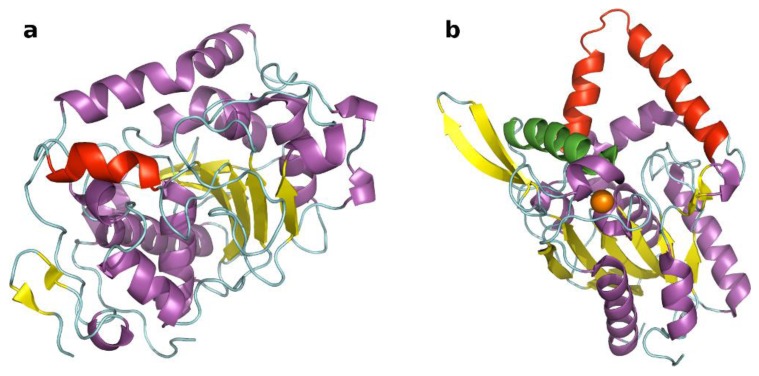
Tridimensional models of CaLB (**a**) (PDB code 1TCA) and PcL (**b**) (PDB code 1YS1). The structures are colored according to their secondary structures; lids are highlighted in red. PcL (**b**) has the second “putative” lid highlighted in green and the Ca^2+^ ion represented as orange sphere.

The present study focuses attention on two lipases that demonstrated different conformational behavior when exposed to a hydrophilic environment, namely lipase B from *Candida antarctica* (CaLB) and *Burkholderia* (*Pseudomonas*) *cepacia* (PcL) (Figure 1). P revious MD simulations indicated that, upon exposure to water, the accessibility of the active site of these lipases is affected only at a minor extent when exposed to polar (e.g., aqueous) media [21].

CaLB is characterized by a small lid [22] and we have previously demonstrated that after 10 ns of MD simulations in explicit water the small lid domain undergoes only some modest conformational changes. Moreover, because of the small size of the lid, there is no closing of the active site. Indeed, the final conformation presents no significant difference in the hydrophobic surface exposed to the bulky aqueous medium [21].

Regarding PcL, MD simulations demonstrated that a β-hairpin domain contributes to the stabilization of a “putative” second lid of PcL in its open conformation by forming two hydrogen bonds between Asn257-Thr224 and between Gln262-Gln215. Only the first one is lost during 20 ns MD in water, so that the upper part of the hairpin moves away from the α-helix but this second putative lid does not change its position considerably and does not occlude the active site [21]. 

**Figure 2:**
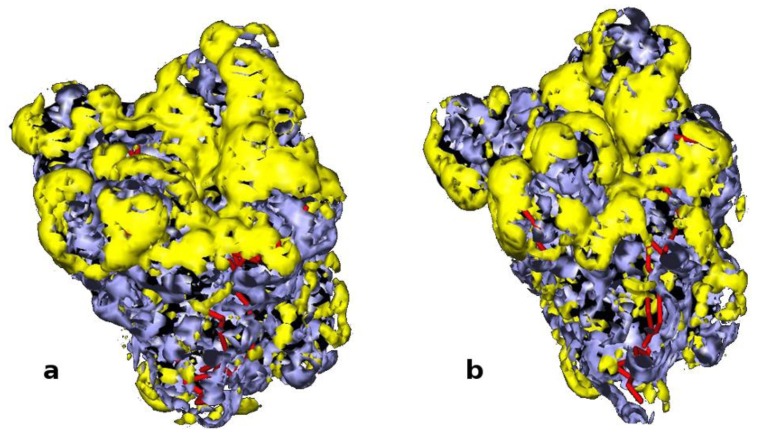
GRID analysis of hydrophobic (yellow) and hydrophilic (blue) surface of PcL. On the left: open conformation corresponding to the crystal structure obtained in the presence of an inhibitor (PDB code 1YS1). On the right: partially closed conformation computed after 20 ns MD simulation in explicit water.

Here, we show (Figure 2) the starting open conformation and the result after 20 ns MD simulations. No significant variation of the superficial domains occurs when the protein is embedded in water and, most importantly, the conformation achieved leads to a partial coverage of the active site. This can be deduced from the mapping of the hydrophobic/hydrophilic areas performed by GRID analysis [23], which reveals how a considerable part of the hydrophobic active site remains exposed to the solvent (Figure 2b).

From the point of view of substrate specificity, lipase B from *Candida antarctica* (CaLB) shows a preference towards short and medium size fatty acids, although it has been investigated extensively also in biodiesel applications [24,25]. It is recognized that the active site of CaLB is situated in the core of the protein, the binding site has a funnel-like shape and highly hydrophobic amino acid residues envelop the cavity inner walls. Consequently, CaLB has, compared to other lipases, a very limited available space in the active site pocket that explains its substrate specificity [26].

Lipase from *Burkholderia cepacia* (PcL), instead, displays a marked specificity for long chain fatty acids and therefore, it appears to be a promising lipase for applications in biodiesel synthesis, as also reported in the literature [27].

In the present investigation, the selection and study of immobilized lipases suitable for biodiesel synthesis was guided by several considerations. Firstly, it must be taken into account that the enzymatic synthesis of biodiesel, unlike oil interesterification, involves the use of relatively high percentages of hydrophilic short chain alcohols that have inactivating effects on enzymes [19]. Moreover, as the reaction proceeds, glycerol is produced. Therefore, the immobilization of lipases for biodiesel synthesis should aim at preventing any detrimental interaction between the biocatalysts and these hydrophilic components, such as aspecific adsorption that might accentuate enzyme inactivation. Moreover, hydrophilic particles generally aggregate in hydrophobic media. On that basis, hydrophobic supports should be preferred.

**Figure 3:**
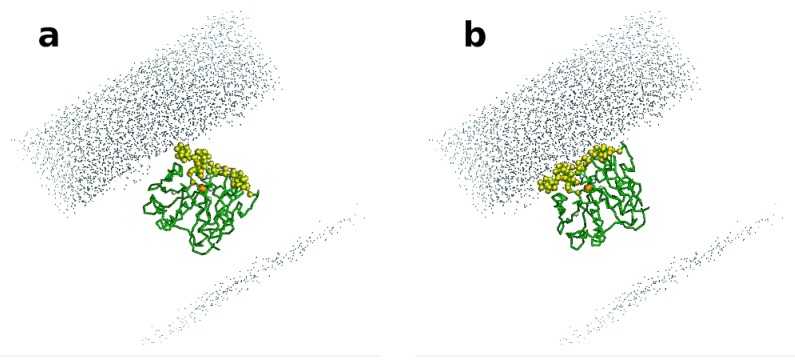
Molecular dynamic (MD) simulation of PcL embedded in an explicit bi-phasic system octane-water. The hydrophobic side of the enzyme, corresponding to the opening of the active site, is highlighted in yellow beads. The octane phase is in grey points in the figure. At the starting point of the simulation (**a**) the water-octane interface is not completely defined; during the simulation the interface was formed (**b**) and the enzyme is oriented with its active site towards the octane part.

However, it must be underlined that lipases recognize and accept very hydrophobic substrates that interact with the lipophilic area corresponding to the opening of the active site. As a continuation of our previous work, we have simulated the behavior of PcL at a water-octane interface (Figure 3) by carrying out a molecular dynamic (MD) simulation using a *coarse grained force field* (MARTINI) [28]. At the beginning of the MD simulation the opening of the active site (yellow dots) is oriented towards the aqueous phase; at the end, the lipase orients the opening of the active site towards the hydrophobic phase. This simulation suggests that the hydrophobic region of the enzyme will be also responsible for the establishment of hydrophobic interactions with hydrophobic supports. Indeed, this feature enables the exploitation of polymeric resins for achieving lipase purification and immobilization in one single step starting from crude enzymatic solutions, where lipases represent the only hydrophobic component [29]. Therefore, such interactions would promote an unfavorable orientation of the enzyme and scarce substrate accessibility. 

On that basis, it seems difficult to define an ideal support for lipase immobilization *a priori*. 

In order to elucidate the effect of the hydrophilic/hydrophobic nature of the carriers, the present study evaluated three different biocatalysts immobilized on highly and medium hydrophobic organic resins specifically developed by Sprin S.p.A. (Trieste, Italy) for biodiesel synthesis (Table 1). Then, the biocatalysts were compared with two preparations immobilized on hydrophilic siliceous carriers, namely diatomaceous earth (Celite). Table 1 reports the characterization of all the biocatalysts using standard assays. 

A commercial formulation of PcL, Lipase PS-IM, was initially considered. It is commercialized by Amano and it consists in lipase PcL adsorbed on diatomaceous earth powder. However, despite the high hydrolytic activity expressed by the enzyme, the preparation was unsuitable for the methanolysis because the hydrophilic powder formed aggregates upon addition of the methanol to the oil. 

**Table 1:** Activity of immobilized lipases tested in the study. CaL = lipase B from *Candida antarctica*. PcL = lipase from *Pseudomonas cepacia.*

Immobilized formulation	Carrier	Hydrophylicity	Particle size	Residualwater ^a^	Synthetic activity	Hydrolitic activity ^d^
				(%)	(U/g dry)	(U/g dry)
PcL-S	Styrenic Porous	---	Beads, 300–500 μm	<5	159 ^b^	463
PcL PS-IM	Siliceous Non porous	++	Powder <20 μm	<5	820 ^b^	15,400
CaL-S	Styrenic Porous	--	Beads, 300–500 μm	<5	3450 ^c^	978
CaL-M	Methacrylic Porous	-	Beads, 300–500 μm	<5	3020 ^c^	490
CaLB on Celite^®^ R-640	Siliceous Porous	+++	Rods 5 × 3 mm	15	2050 ^c^	274

^a^The residual water content in the final immobilized preparation was determined on aluminum plates. A known amount of biocatalyst is dried at 110 °C for 6 h. Solvent content is defined as the % of weight loss after drying. ^b^Synthetic activity: transesterification of vinyl acetate with 1-phenyl-1-ethanol. ^c^Synthetic activity: esterification of lauric acid with 1-propanol. ^d^Hydrolysis of tributyrin.

In light of the aggregation observed using the fine Celite particles, the immobilization of CaLB on hydrophilic carrier was carried out by selecting a commercial porous product, Celite^®^ R-640 (Fluka), which consists in calcined Celite extruded in the form of big porous rods (5 mm high with a diameter of 3 mm). Porosity confers to the immobilization matrix a remarkable ability of adsorbing more than 100% of water (w/w) as well as a cumulative pore volume of 0.8 cm^3^/g [30]. Immobilization was carried out by following a protocol previously developed in our lab for application of biocatalysts in low water media [31]. 

CaL-S and PcL-S are lipases adsorbed on highly hydrophobic styrenic resin whereas CaL-M corresponds to CaLB covalently immobilized on a methacrylic polymer [32]. It appears clear that there is no linear correlation between hydrolytic and synthetic activity. It must be also underlined that no direct comparison is possible between the hydrolytic activity of CaL-S and CaL-M since all enzymes immobilized through physical adsorption undergo partial leaching when suspended in aqueous media and this interfere with kinetic evaluation.

Moreover, the different substrate specificity of CaLB and PcL makes impracticable any direct comparison between the two enzymes. 

### 2.2. Methanolysis of Triolein and Effect of Methanol Concentration

Methanolysis was conducted at 30 °C by using PcL-S (10% w/w of triolein) and by adding three equivalents of methanol obtaining 13% of conversion after 6 h and only 35% after 26 h. The data confirm the severe inactivating effect of methanol. Therefore, in the following experiments, multistep additions of methanol were used, by adding one equivalent molar of methanol at 0, 100 and 200 min. Proton NMR of the crude mixture (see Electronic Supplementary Information, Figure S1) showed the disappearance of the triglyceride peak already after 6 h of reaction. However, upon recycling, the biocatalyst displayed less than 40% of the initial activity. The recycles were carried out by introducing fresh triolein in each cycle and by determining the residual methyl oleate content prior to addition of methanol. Therefore, the PcL-S preparation, although very active, appeared unsuitable for multiple applications in biodiesel synthesis. 

This unexpected behavior might be ascribable to different factors deserving specific investigations, which, however, are out of the scope of the present work. Since PcL displayed such poor efficiency when immobilised on the same support used for CaLB, the cause might reside either in the inherent properties of the protein or in the composition of the crude enzymatic formulation used for the immobilization. PcL is commercialized as a powder containing roughly 1% of protein diluted with cyclodextrins. The effect of this large amount of additives on the final immobilized formulation deserves further attention, although, it must be underlined that purification steps would cause an unacceptable increase of the cost of the biocatalyst. 

One further point of attention is represented by the presence of a calcium ion in the structure of PcL (Figure 1b), which strongly affects the protein stability and this might be lost upon recycling [33].

Finally, a specific effect of some micro-components of triglyceride mixture on PcL stability cannot be excluded. Actually, several compounds generated by atmospheric oxidation of triglycerides, such as hydroperoxides and secondary products, may cause significant lipase inactivation in low water medium. Activity decay is usually time-dependent and shows a non-linear dependence on inactivating compound concentration [34].

When methanolysis was catalyzed by CaLB immobilized on Celite^®^ R-640 (10% w/w of triolein) conversions lower than 5% were observed after 48 h. By adding 2.5% w/w of enzyme at the end of the reaction, there was no improvement in the conversion. It must be underlined that the biocatalyst was able to catalyze tributirin hydrolysis and propil laurate synthesis as shown in Table 1. The data indicate that the stop of reaction is not due to inactivation of enzyme but rather to some other factor connected to the reaction system. Most probably, the hydrophilic and hydrated pores (15% water by weight) of Celite^®^ R-640 do not favor the partition of the oil towards the lipase.

**Figure 4:**
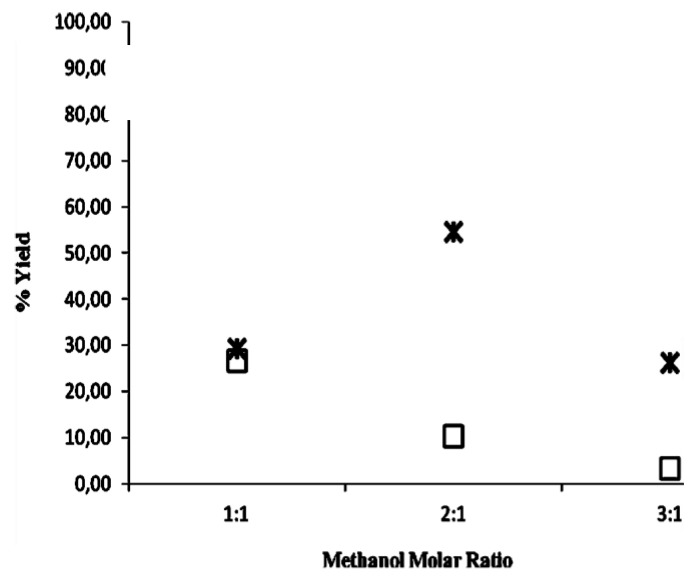
Effect of methanol concentration on CaL-S (star) and CaL-M (square) expressed as the conversion in methyl ester achieved after 25 h. The transesterification of triolein was carried out at 30 °C with molar ratios of 1:1, 2:1 and 3:1. Therefore, theoretical maximum conversions achievable were 33%, 66% and 100%, respectively.

Finally, the two preparations CaL-S and CaL-M immobilized on polymeric organic resins were monitored at different methanol to triolein molar ratios. Figure 4 reports the percentage of activity of the two CaLB formulations, defined as percentage of methyl ester yield evaluated after 25 h of reaction (theoretical maximum conversion in methyl ester is 33%, 66% and 100% for methanol to triolein molar ratio of 1:1, 2:1 and 3:1, respectively). Results indicate that the lipase activity of both CaL-S and CaL-M decreased with the increase of methanol concentration. By using a molar ratio of 3:1 the enzyme inactivation is so fast and severe that the reaction stops after 29% and 5% of conversion, respectively. CaL-S appears more resistant to the inactivating effect of methanol, since a conversion of 54% is achieved at a molar ratio of 2:1. 

**Figure 5:**
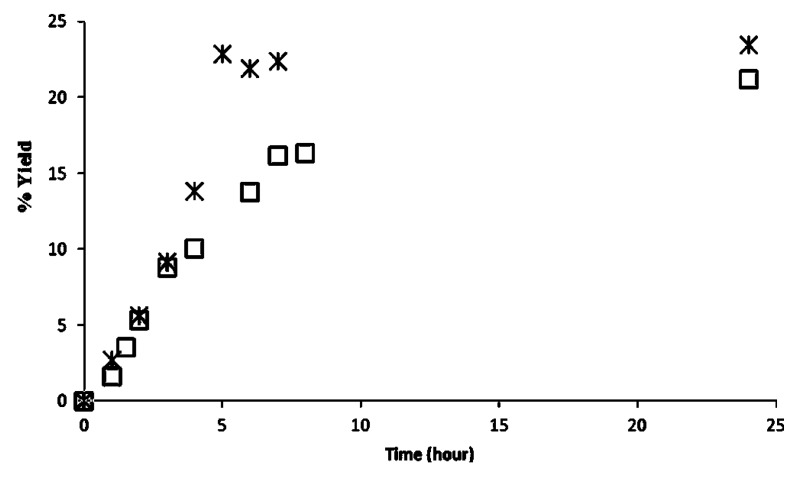
Reaction profiles (24 h) of transesterification of triolein using a 1:1 molar ratio of oil and methanol (30 °C). CaL-S (star) and CaL-M (square). The maximum theoretical yield is 33%.

The different behavior of the two preparations is visible also from the kinetic profile of the reactions carried out at 1:1 molar ratio of triolein and methanol and 10% of biocatalyst (w/w % of triolein) (Figure 5). Apparently, the CaL-M preparation is less efficient even at such low concentration of methanol. In fact, as expected, initial rates are similar since comparable enzymatic units were employed. However the reaction profile of CaL-M indicates that the reaction slows down and, surprisingly, a lower final conversion is achieved, although the thermodynamic equilibrium of the reaction does not depend on the biocatalyst by definition. No further increase of the yield could be achieved upon addition of fresh enzyme and this would suggest that the poorer performance of CaL-M is not due to enzyme inactivation.

**Figure 6:**
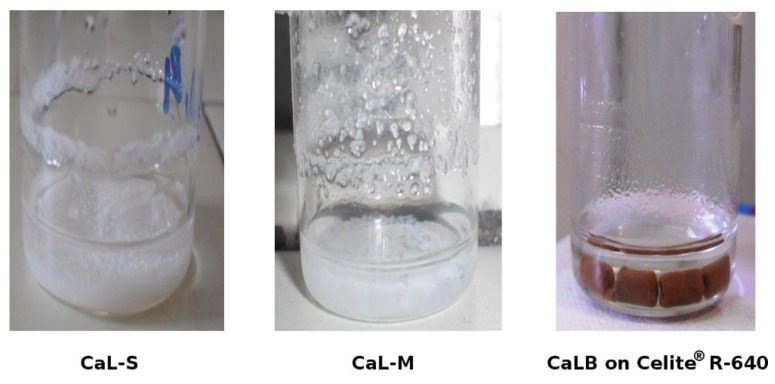
The appearance of the reaction system at the end of the methanolysis using the three preparations of CaLB considered in the study.

CaL-S and CaL-M show a good dispersion of the polymeric supports throughout the reaction with no formation of aggregates. Figure 6 illustrates the appearance of the reaction system at the end of transesterification with stepwise additions of methanol (molar ratio 1:1).

Methanolysis reaction (molar ratio 1:1) using CaL-S gives 24% of conversion after 6h. It must be underlined that we also carried out methanolysis under the same conditions by using *Novozyme 435* obtaining about 10 % of formation of methyl oleate. 

As reported before, the transesterification catalyzed by CaL-S and CaL-M did not achieve a quantitative conversion of the triglyceride. In order to verify whether some methanol was lost during the reaction and the sampling procedure, the transesterification was conducted for 48 h under similar operating conditions but without sampling. The final yield of methyl oleate was 29% for CaL-S but the NMR analysis proved no presence of residual methanol in the reaction medium (see Electronic Supplementary Information, Figure S2). The data suggest that during the transesterification process, some methanol is adsorbed on the immobilization support and this phenomenon is more pronounced in the case of CaL-M, as indicated by the lower conversion achieved. Therefore, the nature of the support appears to play a major role, since an increased local concentration of methanol is expected to be detrimental for the stability of the lipase. This is also in accordance with the lower efficiency of CaL-M observed at higher methanol concentration, as illustrated in Figure 5 above.

The effect of the nature of the immobilization carrier on the partition of hydrophilic components was studied by measuring the water activity of the multiphase reaction systems. Water activity was measured before and after the addition of the immobilized enzymes by means of a hygrometer and after 24 h equilibration as described previously [30,35]. The initial reaction system was a mixture of triolein and methanol with molar ratio 1:1. The values of water activity of the different reaction systems are shown in Table 2.

**Table 2:** Water activity of reaction systems in the presence of different preparations of immobilized CaLB.

Reaction System	Water activity (a_w_)
Reactants mixture	0.64
CaL-S	0.48
CaL-M	0.27
CaLB on Celite^®^ R-640	0.27

All the supports here considered are porous and have the ability to bind a significant amount of water (from 50–100% of their weight) although the residual water (evaluated by weight variation upon drying) resulted to be <5% (w/w) in CaL-S and CaL-M (Table 1). Results show that no immobilized enzyme brings free water to the reaction system that could cause competing hydrolytic reactions [36].

On the contrary, the hydrophilic carriers adsorb some water from the reactants, as indicated by the decrease of a_w_ values, even in the case of Celite R-640, which contains a residual 15% of water in its pores. Significantly, CaL-S, which is the most hydrophobic support, can bind a lower amount of water and, most probably, less methanol as well. 

**Figure 7:**
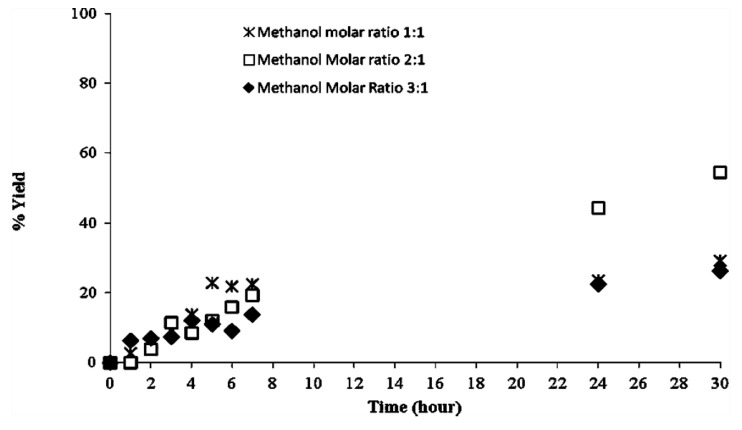
Effect of methanol concentration on CaL-S evaluated at 30 °C by monitoring the transesterification of triolein at different methanol/oil molar ratio.

In order to investigate in more detail the effect of methanol on CaL-S, the kinetic profile of the methanolysis at different concentrations of methanol was studied. Figure 7 shows how, by working with three equivalents of methanol, the inactivating effect is visible already after the first hour of reaction. The residual activity of CaL-S after 1h of reaction at a methanol/triglyceride molar ratio of 3:1 was assayed also by using the method of propil laurate synthesis (see materials and methods). Data indicates that the activity of CaL-S decreases from the initial 3450–1600 (U/g dry), thus confirming the partial inactivation of the enzyme. In the other two cases, the reaction proceeded until the equilibrium was reached and no appreciable variation of conversion was observed by adding fresh enzyme at the end of the reaction (2.5% w/w). This also confirms that the stopping of the reaction was not due to lipase inactivation.

### 2.3. Recyclability of Cal-S

**Figure 8:**
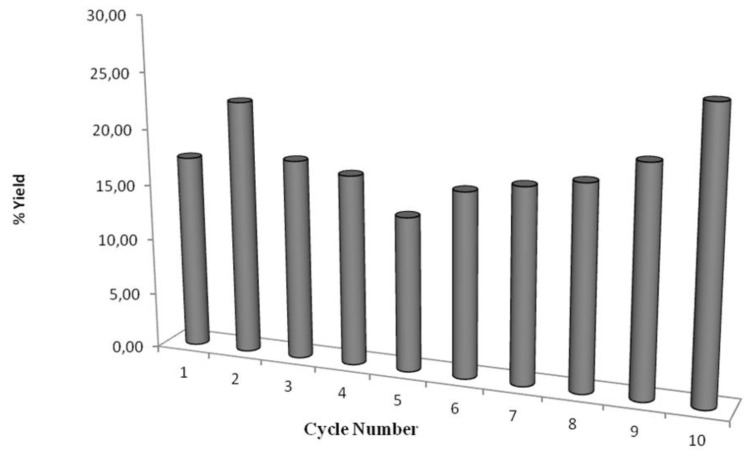
Recyclability of CalB immobilized on styrenic support expressed as the percentage of methyl oleate formed after 4 h at 30 °C in the transesterification of triolein with methanol (1:1 molar ratio).

The stability study was performed using a methanol to oil molar ratio of 1:1 and by stopping the reaction after 4 h in each cycle and measuring the conversion in terms of formation of methyl oleate. Then, the biocatalyst was recovered and reused without further treatment/rinsing by simply introducing fresh triolein and methanol. It must be noted that for each cycle, after introducing fresh triolein, the residual methyl oleate content deriving from the previous cycle was determined prior to the addition of methanol and the starting of the methanolysis. The results indicate that CaLB on styrene is stable after 10 cycles, without any rinsing treatment (Figure 8). Moreover, the biocatalyst has shown its stability even after 22 days of exposure to the reaction conditions. Therefore, no appreciable inhibition due to oil, glycerol or product was detected. Importantly, no aggregation of the particle was observed. The behavior of the preparation indicates that the styrenic resin prevents the undesirable hydrophilic interactions (glycerol and methanol adsorption, protein aggregation, particle aggregation) which are likely to occur in the hydrophobic oils.

Notably, Liù and co-workers reported how CaLB Novozymes 435 undergoes a severe inactivation under comparable conditions, with a loss of roughly 10% of activity after each synthetic cycle [24]. 

### 2.4. Enzymatic Methanolysis of Oil Extracted from Espresso Spent Coffee Ground

As a final step in the investigation of lipase catalyzed methanolysis, the immobilized CaLB was applied to the transformation of a non-edible oil from food waste. The oil was extracted from espresso spent coffee ground properly dried [8] according to the procedure already reported [37]. The oil from spent coffee ground appears as a transparent homogeneous amber colored liquid (see Electronic Supplementary Information, Figure S3). Table 3 illustrates the composition in terms of fatty acids determined by GC-MS and compared to the oil used in the work of Kondamundi. Of course, fatty acids can vary not only as a function of the type of coffee used but also as consequence of the method used for coffee brewing.

**Table 3:** Composition of the oil from espresso spent coffee ground in terms of fatty acids and compared to the oil used in the work of Kondamundi *et al*. [8].

Fatty acids	Oil from spent coffee ground as in [8]	Oil from espresso spent coffee ground
C14	n.d.	n.d.
C16	51.4	58.29
C18	8.3	8.79
C18:1	n.d.	3.41
C18:2	40.3	27.80
C18:3	n.d.	0.09
C20	n.d.	1.61
C22	n.d.	n.d.

The transesterification was carried out using CaL-S on oil from espresso spent coffee ground without pretreatment of free fatty acid and at 30 °C. 

The reaction was monitored via HPLC by evaluating the formation of the methyl esters of the main fatty acids (palmitic, oleic, linoleic, linolenic, stearic, arachidonic). Because of the complexity of the reaction mixture, a semi-quantitative evaluation of the conversion was obtained by summing the areas of these products of the conversion. About 55% of biodiesel yield was achieved by performing one single addition of three equivalents of methanol at the beginning of the reaction whereas multistep additions of one equivalent of methanol (time = 0, 100 min, 200 min) led to 72% conversion after 30 h of reaction. No optimization of the process (e.g., range of time for addition of methanol) was carried out. 

**Figure 9:**
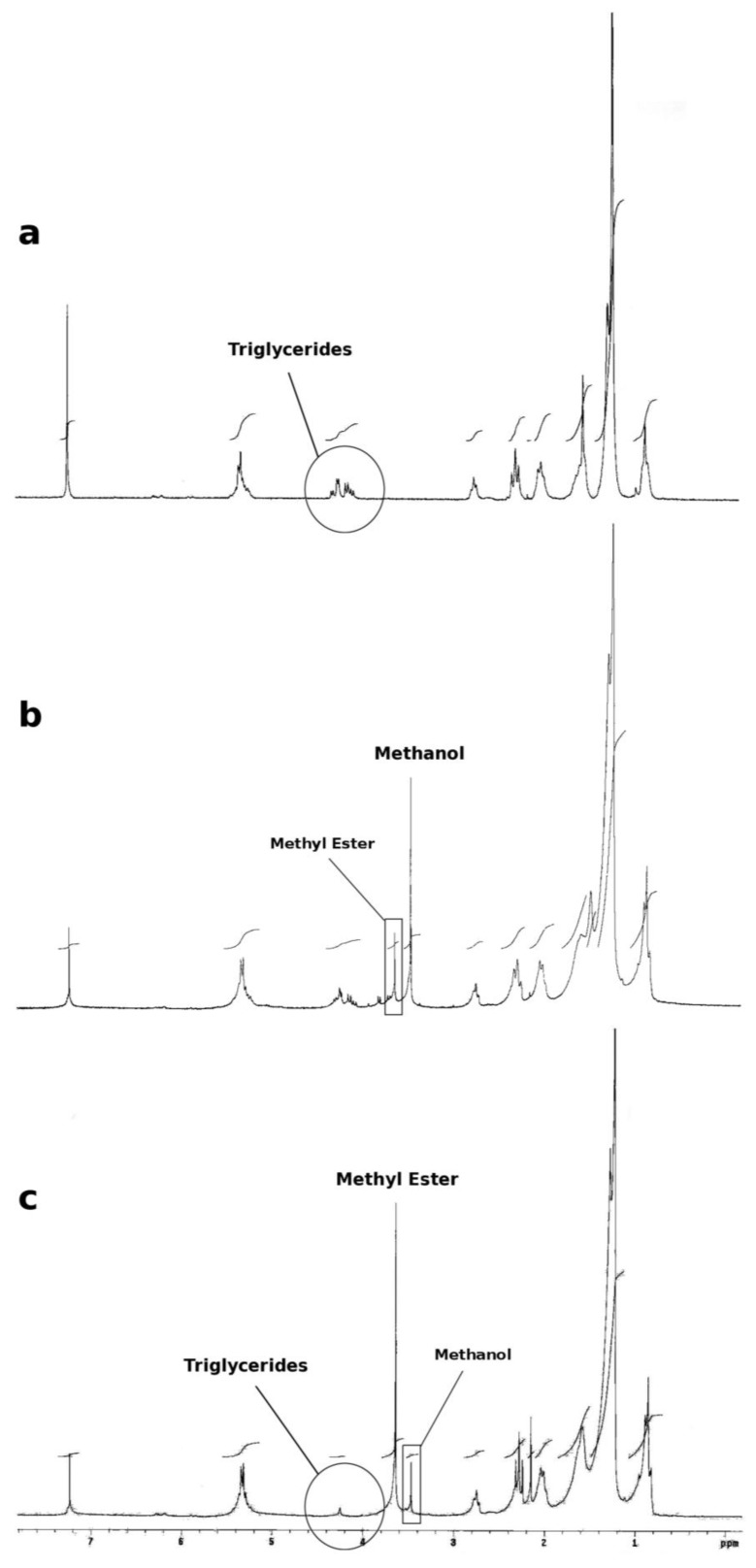
H^1^-NMR spectra of the oil from espresso spent coffee ground (**a**) and of the reaction mixture after 5 h (**b**) and 24 h (**c**).

The decrement of the H^1^-NMR signals relative to the triglycerides was used as a further indicator of the proceeding of the reaction. The area of the protons at 2.5 ppm (signal related to two protons in α-position to carbonyl group of fatty acid) and the area of the protons at 3.6 ppm (signal related to the three protons of methyl esters) indicate that the conversion is nearly quantitative within 24 h (Figure 9).

The GC-MS analysis of the products obtained from the methanolysis (see Electronic Supplementary Information, Figure S4) confirms the formation of the methyl esters of all the fatty acids present in the oil from spent coffee ground, as reported in Table 3. Therefore, CaLB demonstrates to be a versatile biocatalyst, accepting all the different fatty acids present in the oil from spent coffee ground. 

It must be underlined that the chemical (KOH) methanolysis of oil from spent coffee ground reported by Kondamudi required the use of an excess of 40 volumes of methanol and was carried out under reflux at 70 °C. The process described in the present manuscript is carried out at 30 °C and requires no excess of methanol, so that the only co-product of the reaction is the glycerol. Moreover, at the end of the reaction the biocatalyst can be simply filtrated and no acid must be used to neutralize the reaction mixture. 

## 3. Experimental Sections

### 3.1. Enzymes

Native CaLB employed during this study was Lipozyme CalB L from Novozyme (DK). The commercial preparation is constituted by CaLB (11 mg/mL) diluted in a solution of glycerol, sorbitol and salts to stabilize the protein. The pH value of the enzymatic solution is about 4.2.

Native PcL was Lipase PS “AMANO” SD from AMANO (Japan). The commercial preparation is a yellowish lyophilized powder constituted by PcL (about 1%) diluted with dextrins.

Immobilized CaL-M (methacrylic) [32] and CaL-S (styrenic) were kindly donated by Sprin S.p.A. (Trieste, Italy). Organic polymeric resins are in the form of beads with particle size of 300–500 μm. Characterization is reported in Table 1.

The residual water content in the final immobilized preparation was determined on aluminum plates. A known amount of biocatalyst was dried at 110 °C for 6 h. Water content is defined as the % of weight loss after drying.

### 3.2. Immobilization of CalB on Celite^®^R-640

The immobilization was done according to the procedure already reported [31]. The Lipozyme solution was adjusted to pH 8 prior to immobilization. 2.5 mL of enzymatic solution, 1 g of dry Celite^®^ R640 and 12 mL of dry toluene were mixed and kept at 25 °C for one day. The organic solvent was removed and the enzymatic preparation was washed with acetone on a büchner filter under reduced pressure for three times (support/solvent ratio 1/2 w/v). The residual water content was then evaluated as reported above.

### 3.3. Chemicals

Triolein (99%), methyl esters standards and all other chemical reagents are from Sigma Aldrich.

### 3.4. Monitoring the Formation of Fatty acid Methyl Ester (Biodiesel)

Methyl oleate formed from transesterification of triolein was measured by Gilson HPLC equipped with an auto injector. The column was ODS Hypersil SUPERCHROM set at isothermal oven temperature of 54 °C. The detector was UV/VIS-155 with dual wavelength of 200 nm and 210 nm. The mobile phase was 100% Acetonitrile and 0.05% of TFA with flow of 1 mL/min. Biodiesel (methyl esters of fatty acids-FAME) was analyzed by following methods from ref. [10]. Biodiesel yield was calculated by summing the areas of methyl esters present in the reaction mixture and on the basis of calibration curves built up with standards FAME. 

### 3.5. Synthetic Activity of PcL

Activity was determined by transesterification reaction of vinyl acetate with 1-phenyl-1-ethanol, which is a standard synthetic method reported by AMANO Enzymes company. Transesterification reaction was carried out at 25 °C and mixing at 200 rpm. 1-phenylethyl acetate formed during the reaction was measured by Gilson HPLC equipped with an auto injector. The column was ODS Hypersil SUPERCHROM set at isothermal oven temperature of 30 °C. The detector was a Gilson UV/VIS-155 with dual wavelength of 210 nm and 260 nm. The mobile phase was 40% acetonitrile and 0.05% of TFA and 60% H_2_O with a flow of 1 mL/min.

### 3.6. Synthetic Activity of CaLB:

The assay allows the determination of enzymatic activity through the synthesis of propyl laurate formed by enzymatic esterification of lauric acid and 1-propanol. One unit corresponds to the amount of enzyme that produces 1 µmol of propyl laurate in one minute at 55 °C without solvent. In a vial of 20 mL, 1.2 g of lauric acid and 0.36 g of 1-propanol were added. The reaction mixture was thermostated at 55 °C and mixed at 250 rpm in order to allow the melting of lauric acid. A withdrawal of 0.1 mL was diluted in 1.3 mL of hexane. 0.2 ml of this solution was added to 1.6 mL of hexane and analyzed by HPLC (*t* = 0). The reaction was started by adding 40–50 mg of biocatalyst to the main solution and maintained under continuous stirring at 250 rpm. Withdrawals were performed at different times and analysed by HPLC.

### 3.7. Hydrolytics Activity of Lipases

The activity of enzymatic preparations was assayed by following the tributyrin hydrolysis and by titrating, with 0.1 M sodium hydroxide, the butyric acid that is released during the hydrolysis. An emulsion composed by 1.5 mL tributyrin, 5.1 mL gum arabic emulsifier (0.6% w/v) and 23.4 mL water was prepared in order to obtain a final molarity of tributyrin of 0.17 M. Successively, 2 mL of K-phosphate buffer (0.1M, pH 7.0) were added to 30 mL of tributyrin emulsion and the mixture was incubated in a thermostated vessel at 30 °C, equipped with a mechanical stirrer. After pH stabilization, about 50 mg of immobilized protein were added. The consumption of 0.1 M sodium hydroxide was monitored for 15–20 min. One unit of activity was defined as the amount of immobilized enzyme required to produce 1 μmol of butyric acid per min at 30 °C. 

### 3.8. Water Activity

Water activity of the reaction system was determined at 30 °C by using a hygrometer (DARAI-Trieste, Italy) as previously described [30]. Measurements were carried out by sealing the sensor into the open end of 5 mL glasses vials, thermostated at 30 °C, until constant reading. All samples were previously equilibrated for 24 h. The hygrometer was firstly calibrated for 24 h with saturated salt solution of MgCl_2_ (a_w_ = 0.3273), NaCl (a_w_ = 0.7532) and NH_4_NH_2 (_a_w_ = 0.927) using ultrapure water [30,35]. The values of standard salt solution from calibrated hygrometer were 0.39, 0.74 and 0.87.

### 3.9. Monitoring the Methanolysis by HPLC

Methyl esters formed from the transesterification were evaluated by means of a Gilson HPLC equipped with auto injector. The column was ODS Hypersil SUPERCHROM set at isothermal oven temperature of 54 °C. The detector was UV/VIS-155 with dual wavelength of 200 nm and 210 nm. The mobile phase was 100% acetonitrile and 0.05% of TFA with flow of 1 mL/min. Biodiesel (FAME) content and its standards were analyzed by following methods from [38]. Biodiesel yield was calculated based on total area of the methyl ester mixture in the final product. 

### 3.10. Lipase Catalyzed Methanolysis

Transesterification was carried out in 10mL screw capped vials in orbital shaker with temperature control. The temperature was set at 30 °C with shaking rate of 250 rpm. Prior to addition of methanol, the immobilized lipase (10% w/w of oil) was mixed with the feedstock (2 g) for a few minutes. 10 µL samples were withdrawn and analyzed by HPLC. 

### 3.11. Recycling of CaL-S

The stability study was performed using a methanol to oil molar ratio of 1:1, stopping the reaction after 4 hours in each cycle and by measuring the conversion in terms of formation of methyl oleate. Then, the biocatalyst was recovered and reused without further treatment/rinsing by simply introducing fresh triolein and methanol. For each cycle, in order to avoid overestimation of the product concentration, after introducing fresh triolein, the residual methyl oleate content deriving from the previous cycle was determined prior to the addition of methanol and the starting of the new methanolysis cycle. The reactions were performed at 30 °C. 

### 3.12. ^1^H-NMR

Spectra were recorded on a NMR Varian 200 Gemini, operating at 200 MHz. Samples were prepared by diluting 10 µL of the reaction mixture in 700 µL of CDCl_3_.

### 3.13. Extraction of Oil and GC-MS Analysis of Fatty Acids

The spent coffee ground was dried [8] and extracted according to the procedure already reported [37], obtaining yields around 20% (w/w). Characterization was performed according to a method previously described [39] by mean of GC MS Agilent technologies 6890 N and Agilent 5973 MSD as detector. The column was a Zebron ZB-FFAP Capillary Column 60 m × 0.25 mm × 0.25 μm. For the analysis, the temperature raised from 170–235 °C by increasing 2 °C/min. Gas phase was helium at constant flow of 1.3 mL/min. 

### 3.14. Computational Study: Molecular Dynamic Simulations

The molecular dynamic (MD) simulations to analyse the behaviour of lipases upon water exposure were performed using the software GROMACS 4 with the GROMOS-96 53a6 force field. The crystal structure of *Pseudomonas cepacia* lipase (PDB 1YS1) and the crystal structure of *Candida antarctica* lipase B (PDB 1TCA) were implemented in the force field in *gro* file format by using the tool pdb2gmx of the GROMACS software package, which also adds the necessary hydrogen atoms. The proteins were solvated separately with explicit SPC water in a virtual box of 343 nm^3^ each. The molecular dynamic simulations were performed in an NPT environment simulating the temperature of 300 K and keeping the pressure constant (Berendsen thermostat and pressure); the cut-off for electrostatic interactions was set to 1.4 nm and the limit for the Van der Waals interactions set to 1.4 nm. For the minimization procedures, the PME (Particle Mesh Ewald) algorithm was used for the calculation of the electrostatic interactions, setting the limit at 1.0 nm. Before the MD simulation, the systems were minimized at least for 10,000 steps. 

The MD simulation of *Pseudomonas cepacia* lipase at water-octane interface was performed using the software GROMACS 4 with the MARTINI force field. Crystal structure of *Pseudomonas cepacia* lipase (PDB 1YS1) was implemented in the MARTINI force field in *gro* file format by using the necessary scripts available on the MARTINI web site [40] and the DSSP program [41] for the necessary secondary structure definition. The protein was solvated with explicit water-octane 1:1 mixture in virtual cubic boxes of 512 nm^3^. The MD simulation was performed in a NPT environment and in conditions specified above. The time step for integration was set to 4 fs (usually this value is set to 2 fs). Minimization procedures were performed before the MD simulation using a cut-off for electrostatic interactions and by setting the threshold at 1.4 nm. The steepest descendent algorithm was used. Minimization was performed for at least 10,000 steps. 

### 3.15. Computational Study: GRID Mapping of Surface of Proteins

The GRID analysis [32] was performed on the output of the dinamised crystal structure of *Pseudomonas cepacia* lipase (PDB code 1YS1) by choosing a cage big enough to include the whole protein. The grid nodes were set every 0.5 Å. The probes used for the calculation of the molecular interaction fields were DRY (hydrophobic probe) and WATER (H2O probe). Isopotential surfaces were visualized by setting energy values of −0.3 kcalmol^−1^ for the DRY probe and −2.5 kcalmol^−1^ for the WATER probe.

## 4. Conclusions

The present study shows that immobilization plays a major role in determining the efficiency of lipases in the methanolysis of oils. Information on the structure and conformation of lipases provides a rational basis for the selection of biocatalysts and their immobilization. *Ad hoc* immobilization methodologies are necessary for fully exploiting the catalytic potential of lipases while favoring effective mass transfer and partition of substrates/products. 

Lipase B from *Candida antartica* (CaLB) displayed the highest efficiency when immobilized on the most hydrophobic supports (CaL-S). The biocatalyst showed good stability after 10 cycles of reaction in presence of equimolar concentration of methanol. Hydrophilic immobilization supports demonstrated to be less suitable for reactions involving the bulky and hydrophobic triglycerides, most probably because of unfavorable partition phenomena. Moreover, data suggest that these porous carriers are more prone to absorb water or other hydrophilic components such as methanol, which affect negatively the stability of lipases.

The study of methanolysis of oil from spent coffee ground indicates that new types of non-edible oils can be considered for developing competitive and sustainable enzymatic biodiesel synthesis. Immobilized CaLB was able to catalyze the conversion of all different fatty acids present in the raw material. Optimization studies will be required for implementing appropriate feed-batch processes to decrease the reaction time and for the evaluation of various types of oils from different coffee wastes.

## Supplementary Files

Supplementary File 1 Supplementary Information (DOC, 598 KB)

## References

[1] Medina A.R., González-Moreno P.A., Esteban-Cerdán L., Molina-Grima E. (2009). Biocatalysis: Towards ever greener biodiesel production. Biotechnol. Adv..

[2] Mittelbach M., Remschmidt C. (2004). Biodiesel - The Comprehensive Handbook.

[3] Uma B.H., Kim Y.S. (2009). Review: A chance for Korea to advance algal-biodiesel technology. J. Ind. Eng. Chem..

[4] Chisti Y. (2007). Biodiesel from Microalgae. Biotechnol. Adv..

[5] Shah S., Sharma S., Gupta M.N. (2004). Biodiesel preparation by lipase-catalyzed transesterification of jatropha oil. Energy Fuels.

[6] Oliveira L.S., Franca A.S., Camargos R.R.S., Ferraz V.P. (2008). Coffee oil as a potential feedstock for biodiesel production. Bioresour. Technol..

[7] Calabrò V., Ricca E., de Paola M.G., Curcio S., Iorio G. (2010). Kinetics of enzymatic trans-esterification of glycerides for biodiesel production. Bioprocess Biosyst. Eng..

[8] Kondamundi N., Mohapatra S.K., Misra M. (2008). Spent coffee grounds as a versatile source of green energy. J. Agric. Food Chem..

[9] Caetano N.S., Silva V.F.M., Mata T.M. (2012). Valorization of coffee grounds for biodiesel production. Chem. Eng. Trans..

[10] Khan N.A., Brown J.B. (1953). The composition of coffee oil and its component fatty acids. J. Am. Oil Chem. Soc..

[11] Nunes A.A., Franca A.S., Oliveira L.S. (2009). Activated carbons from waste biomass: An alternative use for biodiesel production solid residues. Bioresour. Technol..

[12] Gui M.M., Lee K.T., Bhata S. (2008). Feasibility of edible oil *vs.* non-edible oil *vs.* waste edible oil as biodiesel feedstock. Energy.

[13] Yanagimoto K., Ochi H., Lee K.G., Takayuki S. (2004). Antioxidative activities of fractions obtained from brewed coffee. J. Agricolture Food Chem..

[14] Campo P., Zhao Y., Suidan M.T., Venosa A.D., Sorial G.A. (2007). Biodegradation kitetics and toxicity of vegetable oils triacylglycelols under aerobic conditions. Chemosphere.

[15] Schmid R.D., Verger R. (1998). Lipases: Interfacial enzyme with attractive application. Angew. Chem. Int. Ed..

[16] Mittelbach M. (1990). Lipase-catalyzed alcoholysis of sunflower oil. J. Am. Chem. Soc..

[17] Nielsen P.M., Brask J., Fjerbaek L. (2008). Enzymatic biodiesel production: Technical and economical considerations. Eur. J. Lipid Sci. Technol..

[18] Du W., Xu Y.Y., Liu H., Li Z.B. (2005). Study on acyl migration in immobilized lipozyme TL-catalyzed transesterification of soybean oil for biodiesel production. J. Mol. Catal. B Enzym..

[19] Du W., Xu Y., Liu D., Zeng J. (2004). Comparative study on lipase-catalyzed transformation of soybean oil for biodiesel production with different acyl acceptors. J. Mol. Catal. B Enzym..

[20] Christensen M.W., Andersen L., Husum T.L., Kirk O. (2003). Industrial lipase immobilization. Eur. J. Lipid Sci. Technol..

[21] Ferrario V., Ebert C., Knapic L., Fattor D., Basso A., Spizzo P., Gardossi L. (2011). Conformational changes of lipases in aqueous media: A comparative computational study and experimental implications. Adv. Synth. Catal..

[22] Skjot M., de Maria L., Chatterjee R., Svendsen A., Patkar S.A., Ostergraad P.R., Brask J. (2009). Understanding the plasticity of the alpha/beta hydrolase fold: Lid swapping on the Candida antarctica lipase B results in chimeras with interesting biocatalytic properties. ChemBioChem.

[23] Goodford P.J. (1985). A computational procedure for determining energetically favorable binding sites on biologically important macromolecules. J. Med. Chem..

[24] Xu Y., Du W., Liu D. (2005). Study on the kinetics of enzymatic interesterification of triglycerides for biodiesel production with methyl acetate as the acyl acceptor. J. Mol. Catal. B Enzym..

[25] Watanabe Y., Shimada Y., Sugihara A., Tominaga Y. (2002). Conversion of degummed soybean oil to biodiesel fuel with immobilized *Candida antartica* lipase. J. Mol. Catal. B Enzym..

[26] Anderson E., Larsson K., Kirk O. (1998). One biocatalyst—many applications: The use of Candida antarctica. Biocatal. Biotransform..

[27] Salis A., Pinna M., Monduzzi M., Solinas V. (2005). Biodiesel production from triolein and short chain alcohols through biocatalysis. J. Biotechnol..

[28] Marrink S.J., Tieleman D.P. (2013). Perspective on the Martini model. Chem. Soc. Rev..

[29] Friedrich T., Stuermer R. (2003). Production of immobilized lipase from Pseudomonas and application for enantioselective reactions. U.S. Patent.

[30] Gardossi L., Vulfson E.N., Halling P.J., Holland H. (2001). Immobilization of Enzymes and Control of Water Activity in Low-Water Media: Properties and Applications of Celite R-640 (Celite Rods). Methods in Biotechnology: Enzyme in Non-Aqueous Solvents: Methods and Protocols.

[31] Basso A., de Martin L., Ebert C., Gardossi L., Linda P. (2000). High isolated yields in thermodynamically controlled peptide synthesis in toluene catalysed by thermolysin adsorbed on Celite R-640. Chem. Commun..

[32] Basso A., Braiuca P., Cantone S., Ebert C., Linda P., Spizzo P., Caimi P., Hanefeld U., Degrassi G., Gardossi L. (2007). In silico analysis of enzyme surface and glycosylation effect as a tool for efficient covalent immobilization of CalB and PGA on Sepabeads^®^. Adv. Synth. Catal..

[33] Tanaka A., Sugimoto H., Muta Y., Mizuno T., Senoo K., Obata H., Inouye K. (2003). Differential scanning calorimetry of the effects of Ca2+ on the thermal unfolding of Pseudomonas cepacia lipase. Biosci. Biotechnol. Biochem..

[34] Pirozzi D. (2003). Improvement of lipase stability in the presence of commercial triglycerides. Eur. J. Lipid Sci. Technol..

[35] Ulijn R.V., de Martin L., Halling P.J., Janssen A.E.M., Gardossi L., Moore B.D. (2002). Solvent selection for solid-to-solid synthesis. Biotechnol. Bioeng..

[36] Kaeida M., Samukawa T., Kondo A., Fukuda H. (2001). Effect of methanol and water contents on production of biodiesel fuel from plant oil catalyzed by various lipases in a solvent-free system. J. Biosci. Bioeng..

[37] Ferrari M., Ravera F., De Angelis E., Suggi Liverani F., Navarini L. (2010). Interfacial properties of coffee oils. Colloids Surf. A Physicochem. Eng. Aspects.

[38] Holcapek M., Jandera P., Fischer J., Prokes B. (1999). Analytical monitoring of the production of biodiesel by high-performance liquid chromatography with various detection methods. J. Chromatogr. A.

[39] D’Amelio N., de Angelis E., Navarini L., Schievano E., Mammi S. (2013). Green coffee oil analysis by high-resolution nuclear magnetic resonance spectroscopy. Talanta.

[40] http://md.chem.rug.nl/cgmartini/index.php/home.

[41] Wolfgang K., Sander C. (1983). Dictionary of protein secondary structure: Pattern recognition of hydrogen-bonded and geometrical features. Biopolymers.

