# Supplementary material for: Lipases Immobilization for Effective Synthesis of Biodiesel Starting from Coffee Waste Oils
Source: Biomolecules. 2013 Aug 13;3(3):514–34. doi: 10.3390/biom3030514 (PMC4030945; doi:10.3390/biom3030514)
Supplement: Supplementary File 1 — Supplementary Information (DOC, 598 KB) [file biomolecules-03-00514-s001.doc]

**ELECTRONIC SUPPLEMENTARY INFORMATION**

Lipases Immobilization for Effective Synthesis of Biodiesel Starting from Coffee Waste Oils

Valerio Ferrario ^1^, Harumi Veny ^2^, Elisabetta De Angelis ^3^, Luciano Navarini ^3^, Cynthia Ebert ^1^, and Lucia Gardossi ^1^,*

^1^ Dipartimento di Scienze Chimiche e Farmaceutiche, Università degli Studi di Trieste, Piazzale Europa 1, Trieste 34127, Italy; E-Mails: vferrario@units.it (V.F.); ebert@units.it (C.E.)

^2^ Department of Chemical Engineering, Faculty of Engineering, University of Malaya, Malaysia;
E-Mail: my_harumi@yahoo.com (H.V.)

^3^ illycaffè S.p.A., via Flavia 110, Trieste 34147, Italy;
E-Mails: elisabetta.deangelis@illy.com (E.D.A.); luciano.navarini@illy.com (L.N.)

**Figure S1.** H1 NMR spectra of methanolysis reaction at time = 0 (left) and after 6 h. Methanolysis was catalyzed by PcL-S and one equivalent of methanol was added at time 0, 100 min, 200 min.

**Figure S2.** H^1^ NMR of the product of transesterification catalyzed by CaL-S with a oil:methanol molar ratio of 1:1.

**Figure S3.** Pictures of oil from espresso spent coffee ground (left) and the mixture obtained after enzymatic methanolysis (right).

| 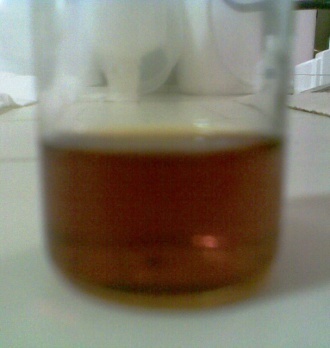 | 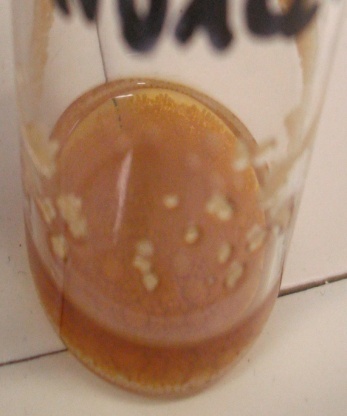 |
| --- | --- |

**Figure S4. GC-MS profiles of fatty acids present in the oil from spent coffee (A) and the products (B) obtained after 30 h of methanolysis catalyzed by CaL-S at 30 °C and with equimolar additions of methanol at time = 0, 100 min and 200 min.**

| 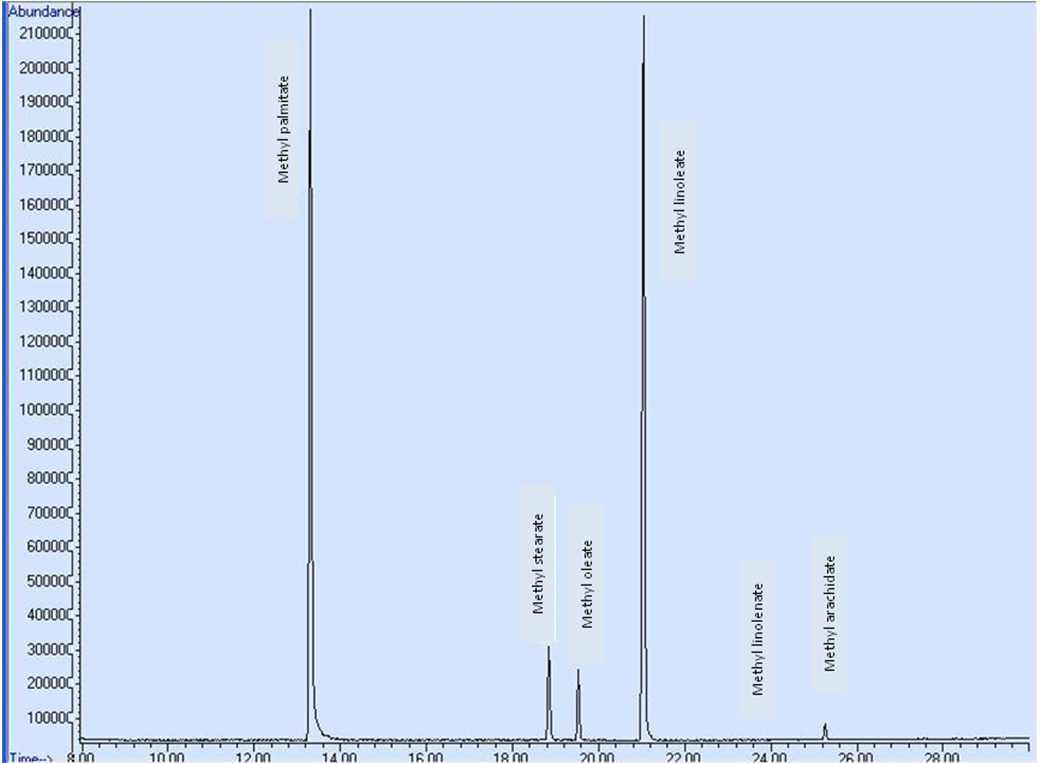 |
| --- |
| (**A**) |
|  |
| (**B**) |
